# Multiphoton NAD(P)H FLIM reveals metabolic changes in individual cell types of the intact cochlea upon sensorineural hearing loss

**DOI:** 10.1038/s41598-019-55329-x

**Published:** 2019-12-11

**Authors:** Paromita Majumder, Thomas S. Blacker, Lisa S. Nolan, Michael R. Duchen, Jonathan E. Gale

**Affiliations:** 1 UCL Ear Institute, University College London, Grays Inn Road, London, WC1X 8EE UK; 2 Research Department of Cell & Developmental Biology, University College London, Gower Street, London, WC1E 6BT UK; 3 Department of Physics & Astronomy, University College London, Gower Street, London, WC1E 6BT UK; 4 Centre for Mathematics and Physics in the Life Sciences and Experimental Biology, University College London, Gower Street, London, WC1E 6BT UK

**Keywords:** Multiphoton microscopy, Biological fluorescence, Cochlea

## Abstract

An increasing volume of data suggests that changes in cellular metabolism have a major impact on the health of tissues and organs, including in the auditory system where metabolic alterations are implicated in both age-related and noise-induced hearing loss. However, the difficulty of access and the complex cyto-architecture of the organ of Corti has made interrogating the individual metabolic states of the diverse cell types present a major challenge. Multiphoton fluorescence lifetime imaging microscopy (FLIM) allows label-free measurements of the biochemical status of the intrinsically fluorescent metabolic cofactors NADH and NADPH with subcellular spatial resolution. However, the interpretation of NAD(P)H FLIM measurements in terms of the metabolic state of the sample are not completely understood. We have used this technique to explore changes in metabolism associated with hearing onset and with acquired (age-related and noise-induced) hearing loss. We show that these conditions are associated with altered NAD(P)H fluorescence lifetimes, use a simple cell model to confirm an inverse relationship between *τ*_bound_ and oxidative stress, and propose such changes as a potential index of oxidative stress applicable to all mammalian cell types.

## Introduction

The World Health Organization estimates that over 5% of the world’s population suffer from a disabling hearing impairment^1^. Acquired sensorineural hearing loss (SNHL) accounts for approximately 90% of these cases^2^, with the most common being the noise-induced (NIHL) and age-related (ARHL) conditions. The vulnerability of cochlear sensory hair cells to trauma and, more recently, the associated loss of cochlear nerve fibres are together believed to be the major causes of SNHL^3^. The contribution of cellular metabolism to this vulnerability is now becoming established^4–10^, guiding attempts at therapeutic intervention^11^. Given the highly heterogeneous cell population present in the sensory organ of the cochlea, the organ of Corti, conventional whole-tissue analyses will fail to reveal which cell types are critical to the pathophysiology. A full understanding of the role of altered metabolism in hearing loss therefore requires experimental approaches capable of interrogating intact cochlear preparations with cellular resolution. Here, we have used fluorescence lifetime imaging microscopy (FLIM) to observe cell-specific metabolic responses in the reduced pool of the metabolic cofactor nicotinamide adenine dinucleotide (NADH) and its phosphorylated analogue NADPH in response to hearing development, maturation and its noise-induced and age-related loss. The measurements motivate the development of a framework for interpreting the results of NAD(P)H FLIM experiments in terms of oxidative stress phenotypes, raising novel questions regarding the metabolic roles of individual cell types in the health and disease of the auditory system.

FLIM extends conventional laser scanning techniques to measure the rate of excited state depopulation at each pixel of an image. This photophysical property, known as the fluorescence lifetime, is altered by changes to the nanoscale local environment of a molecule, thus making FLIM a highly sensitive probe of intracellular biochemistry when applied to the intrinsic fluorescence of living samples^12^. The spectrally-identical autofluorescence of NADH and NADPH is often used for this purpose^13–17^. Both cofactors carry reducing equivalents within the cell but are involved in distinct metabolic pathways^18^. The NAD pool participates primarily in ATP-generating catabolic processes, linking the citric acid cycle to the electron transport chain (ETC) in the mitochondria and regulating the rate of glycolysis in the cytosol. In contrast, NADP is involved in anabolic reactions such as lipid and nucleic acid synthesis and, importantly, in maintaining the glutathione and thioredoxin reductase antioxidant defence systems^15^. The widespread links between altered metabolism and disease have driven the use of NAD(P)H FLIM to investigate these processes in a variety of living cell and tissue models^19–23^.

Free in solution, the fluorescence lifetimes of NADH and NADPH are identical^24^. For either molecule, two species with distinct lifetimes of 0.3 ns and 0.7 ns are observed in aqueous environments, which likely result from alternate configurations of their nicotinamide ring^25^. Inside cells, NAD(P)H emission with a lifetime of 0.4 ns is typically observed, likely reflecting an equilibrium between these two freely-diffusing states, alongside a longer lifetime component which arises through binding of the cofactors to enzymes^15^. NAD(P)H FLIM measurements therefore report three fluorescence decay parameters at each pixel of an image: the shorter lifetime of free NAD(P)H in solution *τ*_free_, the longer average lifetime of the enzyme-bound species present at that pixel *τ*_bound_, and the proportion of the NAD(P)H at that pixel which is enzyme bound *α*_bound_^13^. The quantities *τ*_bound_ and *α*_bound_ have been observed to differ in response to metabolic perturbation and the onset of disease, prompting diagnostic tools to be designed based on time-resolved autofluorescence methods^26^. Indeed, these techniques have previously been applied to investigate the metabolism of the cochlea^19,27^. However, their full potential has yet to be realised as the interpretation of the nanoscale properties reported by the NAD(P)H fluorescence decay in terms of the underlying physiological or biochemical state of the tissue has not yet been unambiguously defined^14–17^.

In our previous work, we sought to understand the biochemical relevance of changes in *τ*_bound_^19^. We demonstrated that this parameter reflects the ratio of [NADPH] to [NADH], as NADPH exhibits a longer fluorescence lifetime than NADH when bound to enzymes within cells. This has allowed us to separately interrogate these functionally-distinct pools using fluorescence techniques for the first time^28–30^. In immature cochlear explant cultures, we measured significantly larger values of *τ*_bound_ in pillar cells, suggesting locally increased [NADPH]/[NADH] ratios, and we showed that this was associated with enrichment of reduced glutathione (GSH) in those cells^19,31^. The present study is a direct extension of that work, now applying NAD(P)H FLIM in the intact cochlea to investigate metabolic contributions to the pathophysiology of NIHL and ARHL.

## Results

### Noise exposure decreases bound NAD(P)H lifetime in both hair cells and pillar (supporting) cells in the organ of Corti

**Figure 1:**
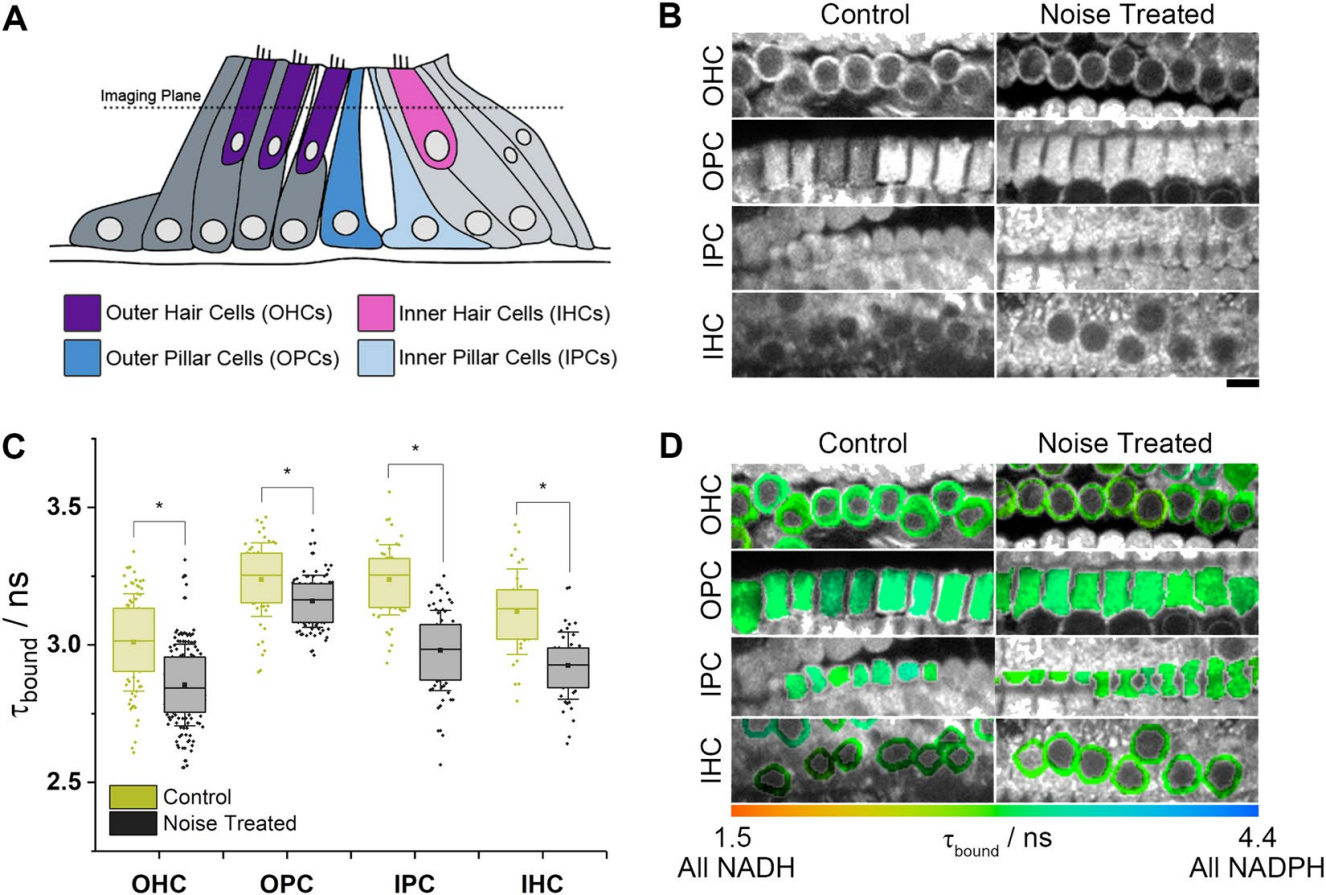
Noise treatment causes a decrease in *τ*_bound_ in both sensory and supporting cells of the organ of Corti. (**A**) Schematic of the organ of Corti, showing the four cell types investigated in this work. (**B**) NAD(P)H fluorescence intensity images of cochlea preparations from three week old (P21–23) mice exposed to mild (TTS) noise treatment (2 hours at 100 dB SPL) compared to control (unexposed) conditions (scale bar 10 µm). (**C**) Mean enzyme bound NAD(P)H fluorescence lifetimes of each cell type (*P < 10^−4^). (**D**) FLIM images colour coded to the mean *τ*_bound_ value in each cellular region of interest.

We compared NAD(P)H FLIM measurements in intact bulla preparations from adult mice (3 weeks old) that had or had not been subject to 100 dB octave-band (8–16 kHz) noise for 2 hours, a noise exposure protocol that elicits a temporary threshold shift (TTS) that persists for at least a day following treatment^32^. We segmented the metabolic responses into the primary functional cells of the organ of Corti shown in Fig. 1A: sensory inner hair cells (IHCs), that convert the mechanical sound stimulus into a nerve signal, sensori-motor outer hair cells (OHCs), which play a role in amplifying this response, and the non-sensory inner and outer pillar cells (IPCs and OPCs) which play structural and trophic roles to support hair cell function^31,33^.

In line with our previous work, we observed that values of *τ*_bound_ under control conditions were significantly (P = 10^−19^) longer in pillar cells than in hair cells, at 3.24(±0.07) ns in OPCs (57 cells across 4 repeats) and IPCs (52 cells) compared to 3.00(±0.10) ns and 3.12(±0.09) ns in OHCs (90 cells) and IHCs (33 cells) respectively. The mild noise exposure caused a significant decrease in *τ*_bound_ in all four cell types (Fig. 1B–D). The extent of the decrease was larger in the IHCs (6%, P = 10^−7^, 46 cells across 4 repeats) and IPCs (8%, P = 10^−17^, 70 cells) than the OHCs (5%, P = 10^−11^, 158 cells) and OPCs (2%, P = 10^−4^, 89 cells).

**Figure 2:**
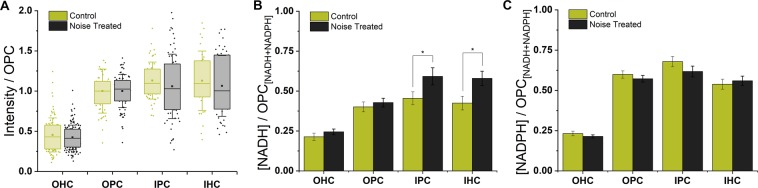
Noise-induced decreases in *τ*_bound_ in IHCs and IPCs result from an increase in NADH in these cells relative to OPCs. (**A**) NAD(P)H fluorescence intensity of each cell type under control and noise-treated conditions, normalised in each image to the mean OPC level. These cells were the most metabolically stable population following noise exposure, based on the smallest changes in NAD(P)H fluorescence decay parameters. (**B,C**) Corresponding NADH and NADPH concentrations, again relative to OPCs, calculated using established procedures^34^ from the fluorescence intensity and decay parameters.

Combining the fluorescence decay parameters with the total intensity changes following noise exposure (Fig. 2A) using established procedures^13,19,34^ allowed the differing responses of the NADH and NADPH pools to be determined. Using this approach, we found that the large decrease in *τ*_bound_ in the IHCs and IPCs upon noise exposure reflected a significant increase in NADH, rather than a decrease in NADPH, relative to the OPCs (Fig. 2B,C).

### Changes in bound NAD(P)H lifetime show distinct patterns between hair cells and pillar cells during maturation of the hearing system

**Figure 3:**
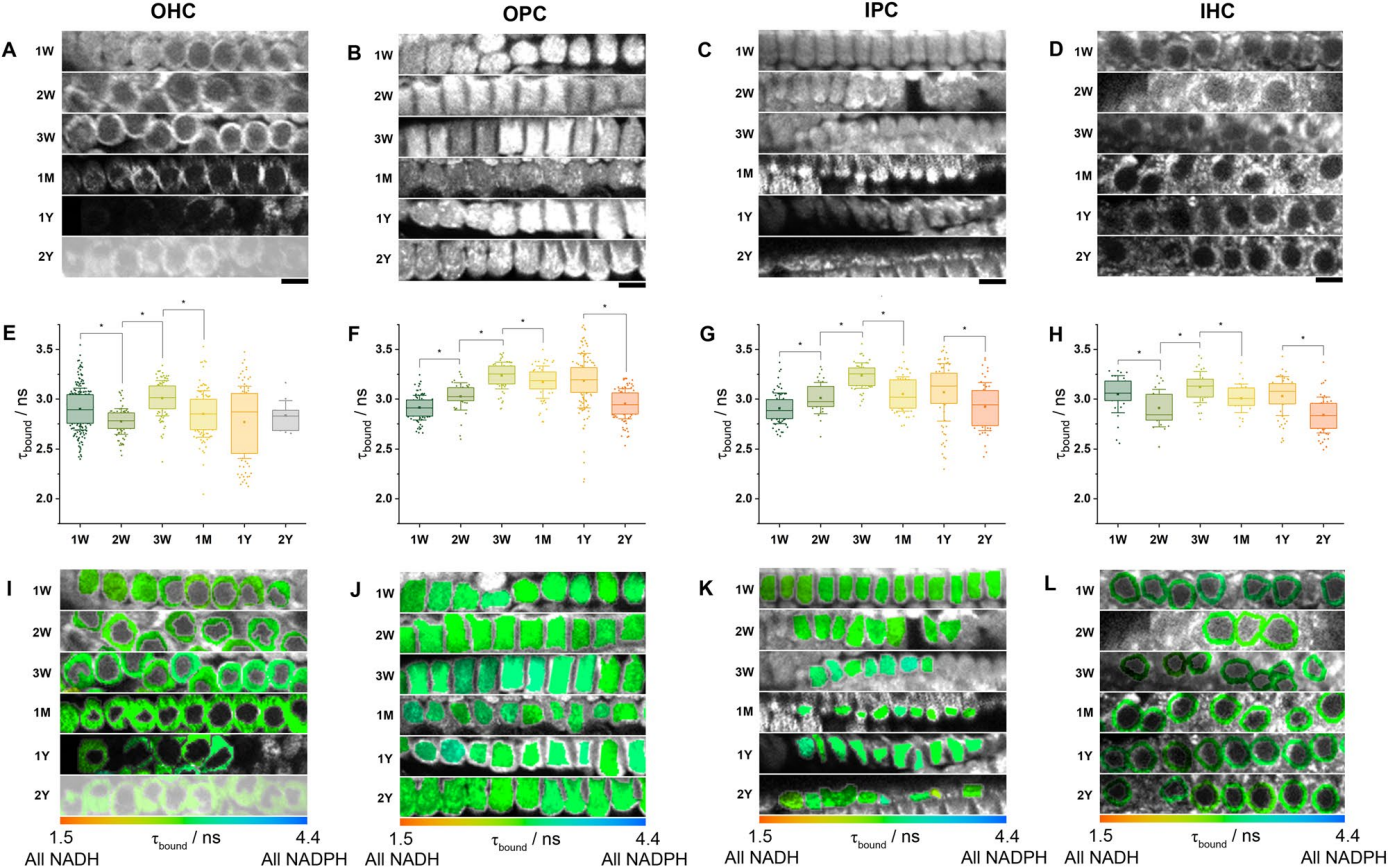
Development and ageing cause distinct patterns of *τ*_bound_ variation in HCs and PCs. (**A–D**) NAD(P)H fluorescence intensity images of each cell type at varying postnatal ages (scale bar 10 µm). (**E–H**) Mean enzyme bound NAD(P)H fluorescence lifetimes *τ*_bound_ (*P < 0.05). (I–L) FLIM images colour coded for the mean *τ*_bound_ value in each cellular region of interest. OHC data is truncated at 1Y due to early-onset hearing loss in C57BL/6 mice.

In rodents at birth, the organ of Corti is not fully mature, supporting cells are not fully differentiated and sensory hair cells are not fully functional. Hearing onset begins after ~12 days, at which point the hair cell synapses are well formed, the endocochlear potential is established and the tunnel of Corti, bounded by the inner and outer pillar cells, has formed^33^. These features become fully mature by one month. While many mouse strains may maintain hearing up to 2 years of age, in the C57BL/6 strain studied here, accelerated cochlear degeneration leads to almost complete deafness by this point^35^, thus providing a well-established model to monitor metabolic changes in the cells of the organ of Corti during post-natal development and with age. We performed NAD(P)H FLIM on cochlear preparations of one week (1 W), two weeks (2 W), three weeks (3 W), one month (1 M), one year (1 Y) and two years (2 Y) of age. These data are shown in Fig. 3, with numerical values available in Supplementary Material Table S1.

At 1 W, in contrast to our previous work in immature rat cochlear explant cultures^19^, the pillar cells did not exhibit the highest *τ*_bound_. At 2.90(±0.08) ns, this parameter was equal in OHCs, OPCs and IPCs, with a significantly larger value in the IHCs at 3.00(±0.20) ns (P = 10^−5^). As hearing developed between 1 W and 2 W, *τ*_bound_ changed in the opposite direction in the hair cells and pillar cells, decreasing to 2.78(±0.06) ns in OHCs (P = 10^−7^) and to 2.91(±0.09) ns in IHCs (P = 0.008) but increasing to 3.03(±0.08) ns in OPCs (P = 10^−6^) and 3.01(±0.08) ns in IPCs (P = 0.002). From 2 W to 3 W, the enzyme-bound lifetime increased in all cell types (P = 10^−5^), establishing the pattern we observed previously of larger *τ*_bound_ in OPCs and IPCs than in the OHCs and IHCs. In contrast, *τ*_bound_ decreased in all cell types between 3 W and 1 M (P = 0.02), indicating possible redox adjustments upon maturation, before remaining constant between 1 M and 1 Y. Development therefore appeared to be accompanied by a significant metabolic switch^36^, before the redox state settled throughout adulthood.

*τ*_bound_ decreased in OPCs, IPCs and IHCs by an average of 6% (P < 0.005) between 1 Y and 2 Y but remained constant in OHCs. However, the validity of the OHC data at this time point in these cells is debatable due to their significant loss by this time in the C57BL/6 strain^37,38^, resulting in only 11 surviving cells being imaged across 10 repeats. For other cell types and ages, the number of cells included in each reported mean are included for reference in Supplementary Material Table S1.

**Figure 4:**
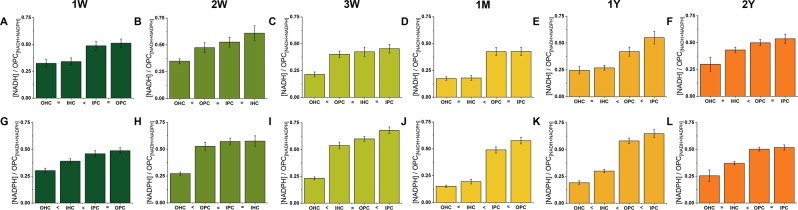
OHCs consistently display the lowest levels of both NADH and NADPH. By combining the NAD(P)H fluorescence intensities and decay parameters using established procedures^34^, internal comparisons between cell types of the absolute NADH (**A–F**) and NADPH (**G–L**) levels could be made at each postnatal age. Differences (<) between cell types were taken as P < 0.05. NADPH levels were typically highest in the pillar cells.

Calculating the relative concentrations of NADH and NADPH in each cell type at each age (Fig. 4), OHCs consistently displayed the lowest NADH levels of the four cell types and the pillar cells the highest. Changes in NADPH levels typically mirrored those of NADH.

### Bound NAD(P)H lifetime may act as an intrinsic indicator of oxidative stress

In accordance with our previous work^19^, changes in *τ*_bound_ have been interpreted as changes in the relative levels of NADH and NADPH. However, it is the NAD +/NADH and NADP +/NADPH redox ratios, rather than the absolute levels of their reduced forms, which are the drivers of the biochemical pathways they regulate^39^. As such, it would be more convenient to be able to understand changes in *τ*_bound_ in terms of the wider metabolic phenotype.

**Figure 5:**
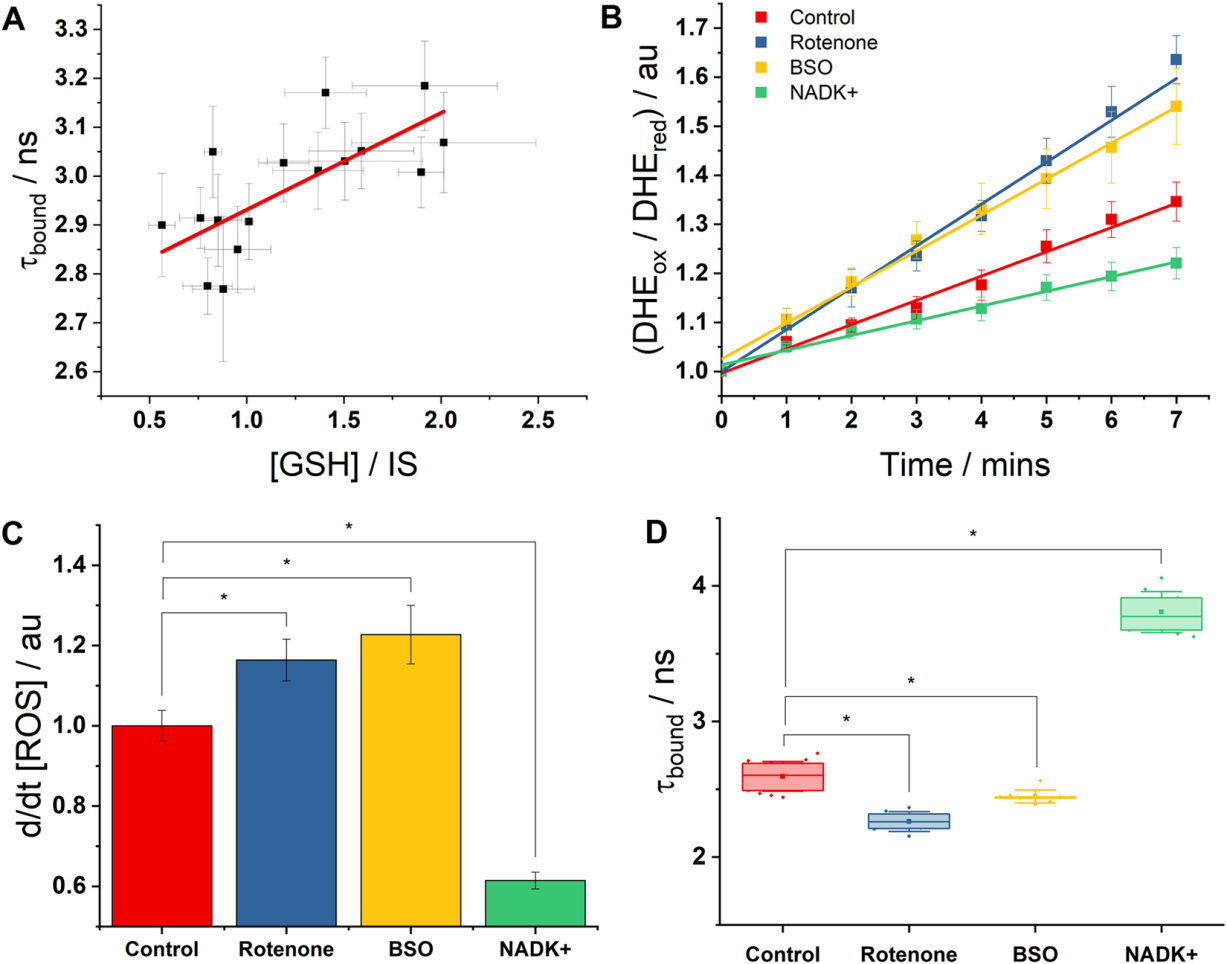
*τ*_bound_ exhibits an inverse correlation with oxidative stress in HEK293 cells. (**A**) Using monochlorobimane (MCB) imaging, the levels of reduced glutathione (normalised to inner sulcus, IS, as previously^31^) in each cell type at 2 W, 1 M and 1Y time-points showed a minor correlation (R^2^ = 0.6) with the corresponding enzyme-bound NAD(P)H fluorescence lifetime data, suggesting that the antioxidative capacity of a system contributes, in part, to the value of *τ*_bound_ measured. (**B,C**) Culturing cells for 24 hours in rotenone (200 nM) or BSO (100 μM) each caused a significant increase in oxidative stress, based on the rate of DHE oxidation. Overexpression of NAD kinase (NADK+) caused this quantity to decrease (*P < 0.05). (**D**) Changes in oxidative stress were mirrored by opposite changes in *τ*_bound_ (*P < 0.001). NADK+ fluorescence lifetime data was taken from previous work^19^.

We previously observed correlations between the high *τ*_bound_ values measured in the pillar cells and increased GSH levels^19^. Corresponding GSH measurements at 2 W, 1 M and 1 Y showed a similar relationship, with an R^2^ correlation coefficient of 0.6 across the four cell types measured (Fig. 5A). The positive correlation suggested that the antioxidant defence level of the system is partially reflected in the measurement of *τ*_bound_. However, as R^2^ was significantly less than one, it is likely that *τ*_bound_ may yet be linked to additional aspects of metabolic state.

A further cellular-level property that may determine the enzyme bound NAD(P)H lifetime can be surmised by considering the contrasting principal roles of NADH and NADPH. The systems for neutralising reactive oxygen species (ROS) inside cells are regulated by NADPH^40^. Given the relationship between [NADPH], [NADH] and *τ*_bound_^19^, the observed positive correlation between *τ*_bound_ and antioxidative capacity is expected. Conversely, high NADH levels drive the production of superoxide, the proximal mitochondrial ROS, by the ETC^41^. With the latter, a negative correlation between *τ*_bound_ and the level of ROS production is predicted. The enzyme bound NAD(P)H lifetime may thus reflect the balance between ROS defence and production, an established metabolic phenomenon known as oxidative stress^15^.

To investigate the possibility of an inverse relationship between *τ*_bound_ and oxidative stress, we performed experiments in simple cell models. Oxidative stress can be increased by increasing the rate of ROS generation or by depleting antioxidative capacity^42^. In HEK293 cells, chronic low-dose rotenone treatment was used to increase the production of ROS by the ETC^43^, and BSO was used to deplete GSH^44^. DHE staining was used to confirm that both treatments increased oxidative stress, with the baseline rate of oxidation increasing by 16(±5)% (P = 0.04) and 23(±6)% (P = 0.02) in the presence of rotenone and BSO respectively (Fig. 5B,C). With both treatments, *τ*_bound_ was observed to decrease, in support of our proposal. Rotenone caused a decrease of 0.33(±0.06) ns (13%, P < 10^−6^) and BSO a decrease of 0.15(±0.05) ns (6%, P = 0.001) as shown in Fig. 5D.

Conversely, to investigate whether increased *τ*_bound_ values correlated with decreased oxidative stress, we repeated the DHE measurements on HEK293 cells in which the enzyme NAD kinase had been overexpressed^45^. We previously observed this perturbation to cause greatly enhanced enzyme bound NAD(P)H fluorescence lifetimes of 3.81(±0.09) ns^19^. In line with our hypothesis, the rate of DHE oxidation in these cells was greatly decreased, by 39(±2)% relative to control (P = 0.001).

NAD kinase overexpression increases the level of NADPH but not NADH^45^, increasing the antioxidative capacity of the cells and the values of *τ*_bound_ measured from them^19^. Using the fluorescence decay parameters and NAD(P)H fluorescence intensity measurements, we calculated the corresponding changes in NADH and NADPH concentrations following rotenone and BSO treatment (Supplementary Material Fig. S5). We observed that *τ*_bound_ decreased following rotenone application due primarily to a 37(±8)% increase in NADH (P = 0.0003), whereas it decreased following BSO due to a 23(±4)% decrease in NADPH (P = 0.002). These data provide the first direct evidence of a convenient relationship between changes in the relative concentration of these two cofactors and the resulting impacts on both the physiological state of oxidative stress and the NAD(P)H fluorescence decay parameter *τ*_bound_.

## Discussion

The sensory hair cells and phalangeal pillar cells are traditionally known as the prime targets of noise exposure^46,47^, although recent data has shown that mild noise exposure can also result in partial loss of auditory nerve fibre (ANF) connections with IHCs^48^. The mechanical activity associated with the sound-induced movements of the organ of Corti is likely to put both the sensory hair cells and structural supporting cells under significant metabolic stress during prolonged noise exposure (i.e. “auditory ageing”). Such metabolic effects are consistent with our observed changes in the enzyme bound NAD(P)H fluorescence lifetime measured in noise-exposed excised bulla preparations in each cell type. This could indicate a common stress mechanism, perhaps as a result of mechanical trauma or due to the propagation of changes in cellular redox state from hair cells to support cells, possibly via intercellular calcium signalling that we have previously described^49–51^. Moreover, intercellular metabolite exchange has previously been demonstrated between different cells in the brain^52^ in the export of GSH by astrocytes for uptake by neurons^53^. This has recently been shown to be mirrored in the cochlea, where supporting cells are thought to contribute to the detoxification of cochlear ROS by releasing GSH through Cx26 hemichannels^54^.

In hair cells, pathological noise exposure caused similar changes as observed around the onset of hearing, i.e. when sound first enters the cochlea^31^, with *τ*_bound_ decreasing between the first and second postnatal weeks. The cochlear data herein are consistent with our proposal that a decrease in enzyme bound NAD(P)H fluorescence lifetime reflects an increase in oxidative stress in cochlear cells. Firstly, as *τ*_bound_ decreased in all cell types from 3 W (maturity) to 2 Y (aged), this correlation would be consistent with theories of ageing based on depletion of antioxidant systems and accumulation of ROS mediated damage^55^. Secondly, the steady increase in *τ*_bound_ in the pillar cells during post-natal maturation of the cochlea is consistent with an increasing expression of antioxidant enzymes observed in brain tissue over the course of postnatal development^56–58^. Finally, as sound exposure causes mechanical and metabolic effects in both hair cells and supporting cells, the acute and marked decrease in *τ*_bound_ in hair cells exposed to sound could reflect the lipid peroxidation, and resulting excess ROS generation, described upon acoustic overstimulation^59^. NAD(P)H FLIM may therefore provide a suitable technique for the assessment of antioxidant therapies for arresting SNHL.

Linking *τ*_bound_ to oxidative stress can also provide further insight into existing NAD(P)H FLIM studies. For example, the frequently-observed shortening of the enzyme bound lifetime in cancer relative to healthy tissue^17,60–62^ could reflect the increase in oxidative stress thought to drive oncogenesis^63^, and our recent observation of diabetic pancreatic islets exhibiting a shorter *τ*_bound_ at low glucose levels than healthy equivalents supports suggestions of increased oxidative stress playing a role in the pathophysiology of this disease^64^. Further, a significant recent advance linking NAD(P)H FLIM measurements to intracellular biochemistry was Schaefer *et al*.’s report of correlations between the mean NAD(P)H lifetime and the pH of the mitochondrial matrix^65^. Specifically, the amplitude-weighted mean lifetime was observed to increase linearly with oxygen consumption rate (OCR), with the constant of proportionality between the two parameters dependent on the matrix pH. Given the definition of the mean lifetime, this constant of proportionality can itself be estimated to be proportional to *τ*_bound_ (see Supplementary Material, Appendix 2). As superoxide production by the ETC is decreased as mitochondrial pH is lowered^66^, our hypothesis would suggest that the mean lifetime would increase with OCR with a larger constant of proportionality at lower mitochondrial pH. This is precisely what Schaefer *et al*. observed, thereby offering a mechanistic explanation for these correlations.

Despite the successes described, the proposed relationship relies on notional roles of enzyme bound NADH and NADPH as contributing to pro- or anti-oxidant pathways only. A potentially confounding factor in this interpretation is in the action of the NADPH oxidases^15^, which produce superoxide by directly reducing molecular oxygen using electrons donated by NADPH. In the cochlea, acute noise exposure upregulates the expression of the NOX1 isoform and this is suggested to contribute to noise-induced cochlear injury by increasing oxidative stress^67^. However, we still observed *τ*_bound_ to decrease under the noise-exposure conditions used herein. An explanation for this could lie in the fast kinetics of the NADPH oxidase enzyme when oxygen is available, in which the cofactor is immediately oxidised by a flavin functional group upon binding, rendering it non-fluorescent and incapable of contributing to the *τ*_bound_ measurement while the enzyme is producing superoxide^68^. Whether this is occurring in the cochlea remains to be tested.

## Conclusion

This work demonstrates the application of NAD(P)H FLIM for monitoring changes in redox-associated metabolism in complex, living tissue samples. We show that metabolic modifications can be identified in different cochlear cell types within 2 hours of mild noise exposure, prior to any overt morphological change of the system, demonstrating the sensitivity of the technique. A decrease in the fluorescence lifetime of enzyme bound NAD(P)H occurred in HCs following noise exposure in both pathological and developmental (onset of hearing) contexts. *τ*_bound_ then reached a peak during cochlear maturation before decreasing during ageing. These observations, combined with the known roles of NADPH and NADH and their relationship to the NAD(P)H fluorescence decay, strongly suggest changes in *τ*_bound_ as a new biomarker for changes in oxidative stress. Such a relationship would open new avenues of research in the NAD(P)H FLIM field, such as in the label-free identification of subsets of cell populations that may be resistant to pro-oxidant therapies^69–72^.

## Methods

### Noise exposure protocol

C57BL/6 mice (P21–23, n = 4) were anesthetized with ketamine (100 mg per kg weight, i.p.) and medetomidine (0.54 mg per kg weight, i.p.) and positioned in a sound proofed booth on a heated pad 45 cm beneath the centre of a speaker (Stage Line PA Horn Tweeter MHD-220N/RD) as described previously^32^. An RX6 processor (Tucker-Davis Technologies, TDT) generated octave-band noise (8–16 kHz) for 2 hours, attenuated (TDT PA5) and amplified (TDT SA2) to a sound pressure level (SPL) of 100 dB. The speaker was calibrated prior to each use to ensure a flat (±2 dB) frequency response over this range. Pedal reflex and breathing rate were checked every 30 minutes. Control animals (n = 4) were not exposed to any additional sound beyond the normal background noise in the animal unit.

### Postnatal auditory bulla preparation

Animals were sacrificed in accordance with the United Kingdom Animals (Scientific Procedures) Act of 1986 using a protocol described previously^31^, approved by the UCL Biological Services Animal Ethics Committee. In addition to the noise exposure experiments, cochleae from 1 week old (P8, n = 3), two week old (P15, n = 3), one month old (P30, n = 4), one year old (P321–381, n = 8) and two year old (P553–721, n = 10) C57BL/6 mice were collected. Auditory bullae were isolated and transferred into Leibovitz’s L15 medium (Thermo Fisher Scientific) and then a window was opened in the apical turn. Reissner’s membrane was peeled away and the stria vascularis removed in order to gain visual access to the organ of Corti^31^. While C57BL/6 mice are well known to have a mutation in the cadherin23 gene (Cdh23^ahl^) that results in an accelerated form of ARHL^37,38^, we nevertheless consider that the relative changes we describe here for the first time are still particularly relevant as an initial internal description of changes in cellular metabolism with age in both sensory and non-sensory cells in the cochlea.

### NAD(P)H FLIM experiments

All experiments were performed using protocols described previously^13,34^ using an upright LSM510 microscope (Carl Zeiss) equipped with a 40× (NA 1.0) water-dipping objective. The basal end of the auditory bullae were secured to the bottom of a 35 mm plastic dish with a small drop of superglue and bathed in L15 medium. NAD(P)H was imaged in the apical coil of the preparation using a Ti:sapphire laser (Chameleon Ultra, Coherent) tuned to 720 nm. Fluorescence was detected by a hybrid photomultiplier tube (HPM-100, Becker & Hickl) after passing through a 435–485 nm emission filter. Emission events were registered by a time correlated single photon counting module (SPC-830, Becker & Hickl). Scanning was performed continuously for 2 minutes with a pixel dwell time of 1.6 μs, resulting in 256 × 256 (125 µm × 125 µm) FLIM images containing between 10^1^ and 10^3^ photons per pixel. 5 × 5 binning (2.5 × 2.5 µm) was applied to increase pixel signal to noise and least squares fitting of the biexponential decay function

1 $$I(t)=I(0)[(1-{\alpha }_{{\rm{bound}}})\exp (-\frac{t}{{\tau }_{{\rm{free}}}})+{\alpha }_{{\rm{bound}}}\exp (-\frac{t}{{\tau }_{{\rm{bound}}}})]$$

was then performed using SPCImage 3.0.8 (Becker & Hickl) using iterative reconvolution with an instrument response function (IRF) recorded from the 460 nm second harmonic generation signal of a potassium dihydrogen phosphate crystal at 920 nm excitation. In this FLIM system, the pulse arrives 4 ns into the time to amplitude converter (TAC), almost a third of the way through the 12.5 ns period between pulses. An incomplete multiexponential model was therefore used and the fitting limits expanded to incorporate the entire trace at each pixel. This avoided wasting the remaining portion of the fluorescence decay recorded in the lead up to the next pulse. A constant (time-uncorrelated) offset was allowed to float at each pixel to account for background fluorescence and the IRF was translated across the time axis to compensate for its dependence on excitation wavelength. To reduce processing time, the IRF position that minimised the ${\chi }_{R}^{2}$ statistic at an arbitrary cytosolic pixel was found and then fixed across the image. Mean parameter values across cellular regions of interest were measured by exporting the data to ImageJ (NIH). NADH and NADPH levels were quantified by combining the fluorescence decay parameters with the total photon counts using previously published procedures^19^. Laser powers at the back aperture of the objective were 17(±1) mW. To account for variations in power at the imaging plane, due either to beam drift or depth of tissue in the beam path, it was necessary to normalise the NAD(P)H concentrations in each image to one cell type in the image window. The outer pillar cells (OPCs) were chosen, being the most metabolically-stable cell type present based on the smallest changes in both *τ*_bound_ and *α*_bound_ following noise exposure.

### Tissue fixation and immunohistochemistry

The viability of all preparations following the experiments was assessed by immunohistochemistry. After fixation in 4% PFA, all bullae preparations were rinsed three times with PBS and incubated in blocking solution (PBS, 10% secondary host antibody serum, 0.5% Triton X-100) for 2 hours^31,73^. The bullae were then washed three times with PBS and incubated for 2 hours at room temperature in blocking solution containing 4′,6′-diamidino-2-phenylindole (DAPI, 1 µM) and phalloidin Alexa Fluor 647 nm (33 nM). The quality of the excised bullae preparations were then evaluated by immunofluorescence (see Supplementary Material Fig. S8). Images were acquired using a Zeiss 510NLO upright confocal microscope using the appropriate excitation wavelengths and emission filters (DAPI 720 nm/435–485BP, phalloidin 633 nm/650LP). The images were acquired at 1.5–2 µm z-intervals using 40x Achroplan (NA 0.8) or 63x Achroplan Vis-IR (NA 1.0) water immersion objectives.

### Glutathione measurements

Monochlorobimane (MCB) passes across the cell membrane and forms a fluorescent adduct when combined with GSH in a reaction catalyzed by glutathione S-transferase. Conjugated GSH-MCB fluorescence can therefore be used as a readout of GSH levels^19,31,44^. After opening, bullae at ages 2 W (n = 3), 1 M (n = 9) and 1Y (n = 8) were incubated in 50 μM MCB (Sigma-Aldrich) for 30 minutes. A subset of this now-expanded dataset has been published previously^31^. GSH-MCB was imaged on a Zeiss 510NLO Axioskop using multiphoton excitation from a Chameleon-XR Ti:Sapphire laser (Coherent) tuned to 780 nm and fluorescence emission was captured using a 435–485 nm bandpass filter. Image stacks were acquired at 2 μm intervals using a 40× (NA 0.8) water immersion objective. All experiments were performed at room temperature (20–23 °C) keeping all confocal imaging parameters constant between experiments.

### Cell culture models of oxidative stress

HEK293 cells were grown in Advanced Dulbecco’s Modified Eagle Medium (DMEM) supplemented with 10% fetal bovine serum, 2 mM GlutaMAX, 100 U ml^−1^ penicillin and 100 mg ml^−1^ streptomycin (all Gibco). Additionally, NADK+ cultures^19^ were grown in the presence of 0.1 mg ml^−1^ G418 (Gibco). Cells were grown as monolayers in sterile 75 cm^2^ tissue culture flasks (Thermo Fisher) in a 37 °C, 5% CO_2_ incubator. For imaging, a 22 mm diameter coverslip was placed in each well of a six well plate (Thermo Fisher) before adding 3 × 10^5^ cells per well. Media was changed after 24 hours, when rotenone (final concentration 200 nM) or buthionine-sulfoximine (BSO, final concentration 100 μM) was added if required. Both stock solutions of the treatments were made up in DMSO, so an equivalent amount of DMSO was added to untreated wells (1 μl in 2 ml of growth media) as a vehicle control. Coverslips were imaged 24 hours later, held in a custom-made stainless steel ring and bathed in DMEM free of phenol red (Sigma) and buffered by 10 mM HEPES.

For oxidative stress assessment, coverslips were loaded with 5 μM dihydroethidium (DHE) for 10 minutes before being imaged on an inverted LSM510 confocal microscope (Carl Zeiss) using a 40× (1.3 NA) oil immersion objective. DHE exhibits blue cytosolic fluorescence until it is oxidised, whereupon its localisation changes to the nucleus and absorption and emission spectra shift to the red^74^. DHE was excited at 351 nm and imaged using 435–485 nm (blue) emission filters. Oxidised DHE was excited at 543 nm and imaged using a 560 nm (red) long pass filter. 512 × 512 images were taken every minute and subsequently analysed in ImageJ. Blue and red fluorescence images were each normalised to their initial values before the blue to red ratio time series was calculated. Three repeats were performed for each treatment and seven repeats were performed under vehicle control conditions. The average red to blue (DHE_ox_/DHE_red_) ratio time series was determined for each condition by taking a mean across repeats and its rate of increase was calculated using a weighted least squares straight line fit in OriginPro 2015 (MicroCal).

### Statistics

Statistical sampling considerations for the FLIM analysis protocol above are described in Supplementary Material Appendix 1. All numerical quantities are reported as mean(±SEM), with n as the number of biological repeats for each condition. Statistical significance (P < 0.05) was judged using unpaired Student’s t-tests. Box and whisker plots use 25^th^ and 75^th^ percentiles for the boxes and standard deviations for the whiskers. Horizontal lines correspond to the median value and the datapoint within the box reflects the mean.

## Supplementary information

Supplementary Information

## Data Availability

Raw datasets available upon request to corresponding authors.
