# Supplementary material for: Multiphoton NAD(P)H FLIM reveals metabolic changes in individual cell types of the intact cochlea upon sensorineural hearing loss
Source: Sci Rep. 2019 Dec 11;9:18907. doi: 10.1038/s41598-019-55329-x (PMC6906381; doi:10.1038/s41598-019-55329-x)
Supplement: Supplementary file 1 — Supplementary Information [file 41598_2019_55329_MOESM1_ESM.doc]

***SUPPLEMENTARY MATERIAL***

# Multiphoton NAD(P)H FLIM reveals metabolic changes in individual cell types of the intact cochlea upon sensorineural hearing loss

Paromita Majumder^1,*,†^, Thomas S. Blacker^2,3,4,*,†^, Lisa S. Nolan^1^, Michael R. Duchen^2^, Jonathan E. Gale^1,2^

^1^UCL Ear Institute, University College London, Grays Inn Road, London WC1X 8EE, UK.

^2^Research Department of Cell & Developmental Biology, University College London, Gower Street, London WC1E 6BT, UK.

^3^Department of Physics & Astronomy, University College London, Gower Street, London WC1E 6BT, UK.

^4^Centre for Mathematics and Physics in the Life Sciences and Experimental Biology, University College London, Gower Street, London WC1E 6BT, UK.

# ^†^Corresponding authors: paromita.majumder@gmail.com, t.blacker@ucl.ac.uk

# ^*^These authors contributed equally to this work

|  | Biological Repeats | $\tau_{\text{bound}}$ / ns | | | |
| --- | --- | --- | --- | --- | --- |
|  |  | OHC | OPC | IPC | IHC |
|  |  | [Number of cellular ROIs] | | | |
| 1W | 3 | 2.9(±0.1)  [224] | 2.91(±0.06)  [89] | 2.91(±0.08)  [77] | 3.05(±0.09)  [31] |
| 2W | 3 | 2.78(±0.06)  [87] | 3.03(±0.08)  [44] | 3.01(±0.08)  [32] | 2.91(±0.09)  [24] |
| 3W | 4 | 3.0(±0.1)  [90] | 3.24(±0.07)  [57] | 3.24(±0.07)  [52] | 3.12(±0.09)  [33] |
| 3W-noise | 4 | 2.85(±0.07)  [158] | 3.16(±0.05)  [89] | 2.98(±0.08)  [70] | 2.92(±0.07)  [46] |
| 1M | 4 | 2.85(±0.09)  [107] | 3.17(±0.07)  [55] | 3.05(±0.08)  [53] | 3.01(±0.07)  [27] |
| 1Y | 8 | 2.8(±0.1)  [83] | 3.18(±0.09)  [140] | 3.1(±0.1)  [75] | 3.03(±0.08)  [52] |
| 2Y | 10 | 2.83(±0.09)  [11*] | 2.95(±0.05)  [101] | 2.92(±0.08)  [48] | 2.84(±0.06)  [55] |
|  |  | HEK cells [Number of fields of view] | | | |
| Control | 5 | 2.60(±0.04) [14] | | | |
| Rotenone | 3 | 2.26(±0.04) [8] | | | |
| BSO | 3 | 2.45(±0.03) [9] | | | |
| NADK+ | 3 | 3.81(±0.09) [9] | | | |

**Table S1:** Summary of $\tau_{\text{bound}}$ values and numbers of regions of interest (ROIs) for each experimental condition. The low number of OHCs observed in the 2 year old animals (*) reflects the accelerated hearing loss in the C57BL/6 strain. n biological repeats allow 2n preparations per animal in the cochlea experiments. For cell culture experiments, biological repeats refer to the number of separate coverslip cultures.

**Appendix 1:** Sampling considerations for FLIM

In our previous work, numerical simulations demonstrated that reliable fitting of a bi-exponential model required fluorescence decay measurements with 200 or more counts in the peak channel^1^. With average count rates of around 10^5^ photons per second spread across a 256x256 image, achieving this signal level would require acquisition times of approximately an hour per image. We therefore instead binned 24 surrounding pixels to increase the signal at each pixel, preserving the integrity of the tissue, at the expense of spatial resolution, by reducing the acquisition time to two minutes.

Poisson noise inherent in the TCSPC method will cause pixel to pixel variations in the reported lifetime values, even for a homogeneous fluorescent sample. We carried out numerical simulations to assess the impact of this on our experimental design. Synthetic datasets were constructed in MATLAB by sampling the function

| $I\left(t\right)=I\left(0\right)\left[\left(1-\alpha_{\text{bound}}\right)\exp\left(-\frac{t}{\tau_{\text{free}}}\right)+\alpha_{\text{bound}}\exp\left(-\frac{t}{\tau_{\text{bound}}}\right)\right]$ | (S1) |
| --- | --- |

at intervals of 48.8ps, representing the bin width of our TCSPC setup (256 bins spread across 12.5 ns). , and were set to 0.2, 0.4 ns and 3 ns respectively. The dataset was then convolved with the measured instrument response function of the system and scaled to a given *I*(0) value, where *I*(0) is the number of photons in the peak channel of the decay. Poisson noise was added to each bin using the poissrnd() function. Non-linear least squares fitting was then performed using the FLUOFIT package^2^ and the extracted value stored in memory. This process was performed 1000 times at each *I*(0) value. The standard deviation of values are shown in Figure S1, where the values closely correspond to the *I*(0)^-1/2^ dependence expected from a Poisson process.

The inherent pixel-to-pixel uncertainty in the lifetime values reported by FLIM motivated us to extract data from the images by taking mean values across regions of interest drawn over the entirety of the cytoplasm of each cell. Mitochondria (identified as punctate regions of bright NAD(P)H fluorescence) could not be clearly recognised at the magnifications permitted by the bulla preparation, so the values reported reflect a mixture of cytosolic and mitochondrial signals. However, we have previously identified very little difference in the fluorescence decay parameters of these two compartments^1^, so we expect this spatial averaging to have little effect. In contrast, we have previously shown that NAD(P)H fluorescence in the nucleus does vary significantly from the mitochondria and cytosol^1^. This region can be clearly identified as a darker, circular area of NAD(P)H fluorescence so was excluded from ROIs.

Cellular regions of interest were typically between 100 and 400 pixels in size. To understand how the mean values across these regions of interest would reflect the underlying “true” lifetime, we performed further numerical simulations. 10,000 synthetic fluorescence decay datasets were generated using the same $\alpha_{\text{bound}}$, and values as above, with 500 counts in the peak channel to reflect the typical signal levels we obtained experimentally. Each dataset was fit to a biexponential decay model as above, and the reported value recorded. The effect of ROI size on measurement accuracy was then assessed by taking subsets of these values of differing sizes, from 1 to 500 pixels. The mean value of each artificial ROI was then recorded, repeating 1000 times for each ROI size. The results are shown in Figure S2, where it can be seen that as the ROI size increases, the spread of values reported decreases. For a 100 pixel ROI area, the reported values have a standard deviation of 0.5%. In contrast, from the initial simulations, the uncertainty in of a single decay measurement with 500 counts in the peak channel is approximately 5%.

The above simulations show that averaging over a region of interest negates the effect of Poisson noise. Implicit in this approach is the assumption that the metabolic state is constant across the cell, and that all pixel-to-pixel variations in lifetime within the ROI are due to Poisson noise alone. Cell-to-cell variations in lifetime would then reflect the biological variation expected in a heterogeneous tissue such as the cochlea, allowing statistical hypothesis tests to be performed to evaluate the significance of differences between the fluorescence decay parameter values of each cell group.


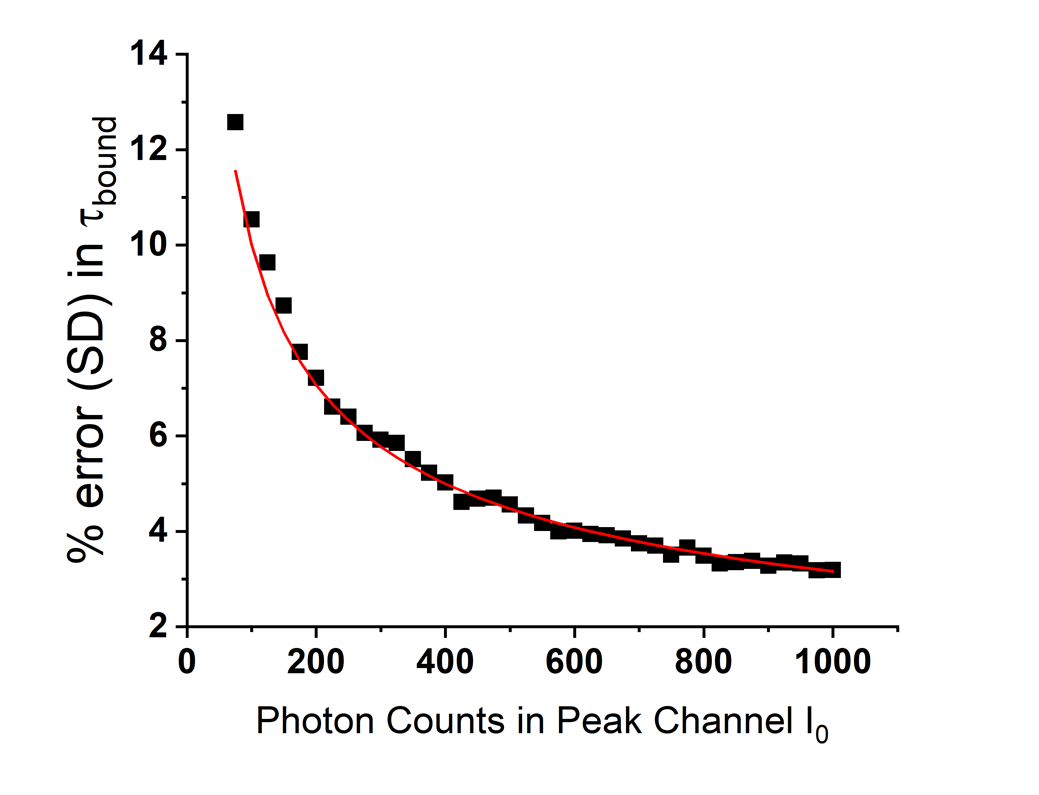


**Figure S1:** Least-squares fitting of synthetic NAD(P)H fluorescence decay datasets revealed that the spread of $\tau_{\text{bound}}$ values reported across 1000 repeats at each I_0_ value clearly followed the *I*(0)^-1/2^ dependence expected from a Poisson process.


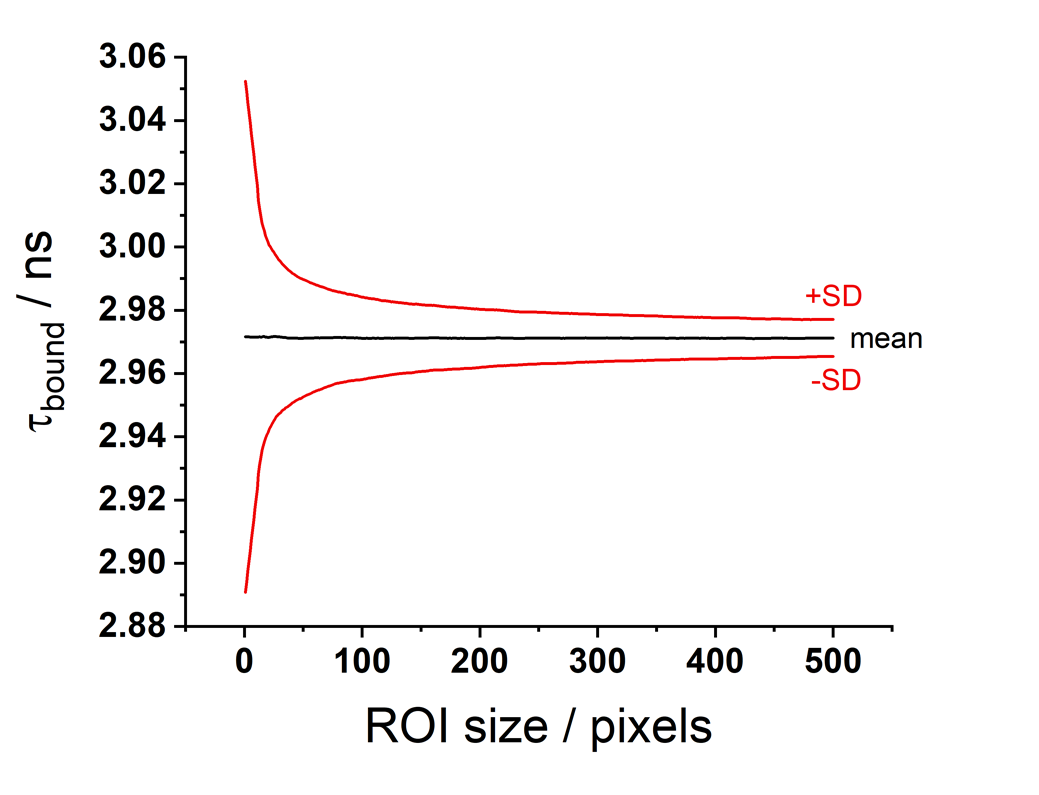


**Figure S2:** Synthetic ROIs were constructed containing increasing numbers of synthetic datasets, each generated using identical underlying fluorescence decay parameters but regenerated Poisson noise. The mean $\tau_{\text{bound}}$ value in each synthetic ROI was recorded and the process was repeated 1000 times for each ROI size. The spread of $\tau_{\text{bound}}$ values reported decreases rapidly between ROIs of 1 and 50 pixels. For 100 pixel ROIs, the minimum area applied in this work, the spread of values extracted was only 0.5%.

**Appendix 2:** Linking NAD(P)H FLIM, redox state, matrix pH and oxidative stress

Under conditions where the concentration of enzymes can be assumed to remain constant, simple enzyme kinetics predicts that $\alpha_{\text{bound}}$ is inversely related to changes in the concentration of NAD(P)H. This can be seen by considering the usual definition of the dissociation constant $K_{D}$, given by,

| $K_{D}=\frac{\left[\text{NAD(P)H}\right]\left[\text{E}\right]}{\left[\text{E}\cdot\text{NAD(P)H}\right]}$ | (S2) |
| --- | --- |

where $\left[\text{NAD(P)H}\right]$ and $\left[\text{E}\right]$ are the concentrations of NAD(P)H and enzymes that are not in a complex and $\left[\text{E}\cdot\text{NAD(P)H}\right]$ is the concentration of enzyme-bound NAD(P)H. In this context, the total concentrations of NAD(P)H and enzymes, $\left[\text{NAD(P)H}\right]_{0}$ and $\left[\text{E}\right]_{0}$, remain constant, giving,

| $\left[\text{NAD(P)H}\right]_{0}=\left[\text{NAD(P)H}\right]+\left[\text{E}\cdot\text{NAD(P)H}\right]$ | (S3) |
| --- | --- |
| $\left[\text{E}\right]_{0}=\left[\text{E}\right]+\left[\text{E}\cdot\text{NAD(P)H}\right]$ | (S4) |

Equation S2 can then be rewritten,

| $K_{D}=\frac{\left(\left[\text{NAD(P)H}\right]_{0}-\left[\text{E}\cdot\text{NAD(P)H}\right]\right)\left(\left[\text{E}\right]_{0}-\left[\text{E}\cdot\text{NAD(P)H}\right]\right)}{\left[\text{E}\cdot\text{NAD(P)H}\right]}$ | (S5) |
| --- | --- |

Rearranging this gives the quadratic expression,

| $\left[\text{E}\cdot\text{NAD(P)H}\right]^{2}-\left(K_{D}+\left[\text{NAD(P)H}\right]_{0}+\left[\text{E}\right]_{0}\right)\left[\text{E}\cdot\text{NAD(P)H}\right]+\left[\text{NAD(P)H}\right]_{0}\left[\text{E}\right]_{0}=0$ | (S6) |
| --- | --- |

Solving using the quadratic formula gives,

| $\begin{matrix} \left[\text{E}\cdot\text{NAD(P)H}\right] \\ =\frac{K_{D}+\left[\text{NAD(P)H}\right]_{0}+\left[\text{E}\right]_{0}\pm\sqrt{\left(K_{D}+\left[\text{NAD(P)H}\right]_{0}+\left[\text{E}\right]_{0}\right)^{2}-4\left[\text{NAD(P)H}\right]_{0}\left[\text{E}\right]_{0}}}{2} \end{matrix}$ | (S7) |
| --- | --- |

$\alpha_{\text{bound}}$ can therefore be written,

| $\begin{matrix} \alpha_{\text{bound}}=\frac{\left[\text{E}\cdot\text{NAD(P)H}\right]}{\left[\text{NAD(P)H}\right]_{0}} \\ =\frac{1+\frac{K_{D}}{\left[\text{NAD(P)H}\right]_{0}}+\frac{\left[\text{E}\right]_{0}}{\left[\text{NAD(P)H}\right]_{0}}-\sqrt{\left(1+\frac{K_{D}}{\left[\text{NAD(P)H}\right]_{0}}+\frac{\left[\text{E}\right]_{0}}{\left[\text{NAD(P)H}\right]_{0}}\right)^{2}-4\frac{\left[\text{E}\right]_{0}}{\left[\text{NAD(P)H}\right]_{0}}}}{2} \end{matrix}$ | |
| --- | --- |
|  | (S8) |

where the negative root has been taken to ensure $0\leq\alpha_{\text{bound}}\leq1$.

Equation S8 is plotted as a function of $\left[\text{NAD(P)H}\right]_{0}$ in Figure S3 for $K_{D}=1$ and varying values of $\left[\text{E}\right]_{0}$, demonstrating that for a given level of NAD(P)H-binding enzymes, $\alpha_{\text{bound}}$ decreases as the amount of reduced NAD(P)H increases. This relationship is experimentally verified when applying acute pharmacological perturbations to raise or lower NAD(P)H levels; $\alpha_{\text{bound}}$ increases upon oxidising the mitochondrial NAD pool by applying an uncoupler^1^ and decreases upon reducing the NAD pool by inhibiting the ETC^3^. These results have previously been used as justification for interpreting differences in the fraction of enzyme bound NAD(P)H as differences in cellular redox balance^4^, and subsequently metabolic state^5^. However, this interpretation is likely to be invalid^6^, as Equation S8 also depends on the abundance of enzyme binding sites. While expression patterns of NAD(P)-linked dehydrogenases may not change over the timescale of application of a metabolic inhibitor, they are likely to differ between cell types or in response to the onset of disease^7^. NAD and NADP biosynthesis is also likely to differ^8^, complicating the assumption implicit in this interpretation of $\alpha_{\text{bound}}$ that changes in NAD(P)H reflect only a change in redox state, and not a change in the size of the total NAD(P) pool.

The present work does not attempt to assign physiological significance to the changes in $\alpha_{\text{bound}}$ observed (included for reference in Figures S4-S7) but explores the functional consequences of the relationship between the relative abundances of NADPH and NADH and the value of $\tau_{\text{bound}}$ measured in a cell or tissue that was developed in our previous study^1^. Since this work, a significant report by Schaefer et al. has highlighted the role of the pH of the mitochondrial matrix in determining the mean lifetime of NAD(P)H fluorescence^9^. The mean lifetime is given by,

| $\left\langle\tau\right\rangle=\left(1-\alpha_{\text{bound}}\right)\tau_{\text{free}}+\alpha_{\text{bound}}\tau_{\text{bound}}$ | (S9) |
| --- | --- |

Schaefer et al. gradually altered the redox state of the mitochondrial NAD pool by applying increasing concentrations of an uncoupler or complex I inhibitor, and $\left\langle\tau\right\rangle$ was observed to increase linearly with oxygen consumption rate with the constant of proportionality between the two parameters dependent on the matrix pH. Increases in the oxygen consumption rate (OCR) will decrease the levels of reduced NAD(P)H which, based on Equation S8, will increase $\alpha_{\text{bound}}$. We can therefore propose that the change in mean lifetime with OCR measured by Schaefer et al. is proportional to the change in mean lifetime with $\alpha_{\text{bound}}$, obtained by differentiating Equation S9,

| $\frac{\partial\left\langle\tau\right\rangle}{\partial\text{OCR}}\propto\frac{\partial\left\langle\tau\right\rangle}{\partial\alpha_{\text{bound}}}=\tau_{\text{bound}}-\tau_{\text{free}}=k$ | (S10) |
| --- | --- |

If we unite this relationship with the hypothesis we develop in the present study, situations in which oxidative stress is lower would result in a higher $\tau_{\text{bound}}$ and therefore a larger proportionality constant $k$ between the mean lifetime and the redox state. Schaefer et al. observed this proportionality constant to be larger when the mitochondrial matrix pH was lower. Under these conditions, superoxide production by the ETC is decreased^10^, lowering oxidative stress. The conclusions of this significant work can therefore be explained mechanistically by the hypothesis we develop here.


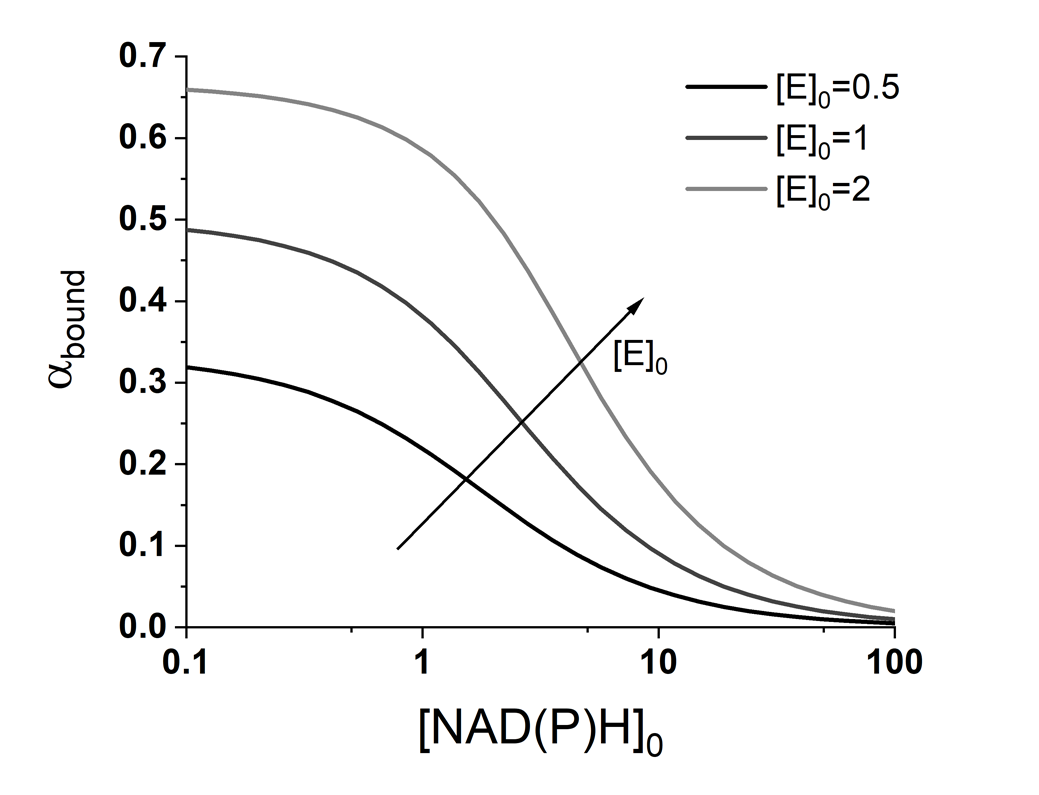


**Figure S3:** Plots of a simple enzyme binding model for $\alpha_{\text{bound}}$ (Equation S8). The fraction of enzyme bound NAD(P)H decreases with increasing reduced NAD(P)H, but the precise value depends on the concentration of enzymes present.


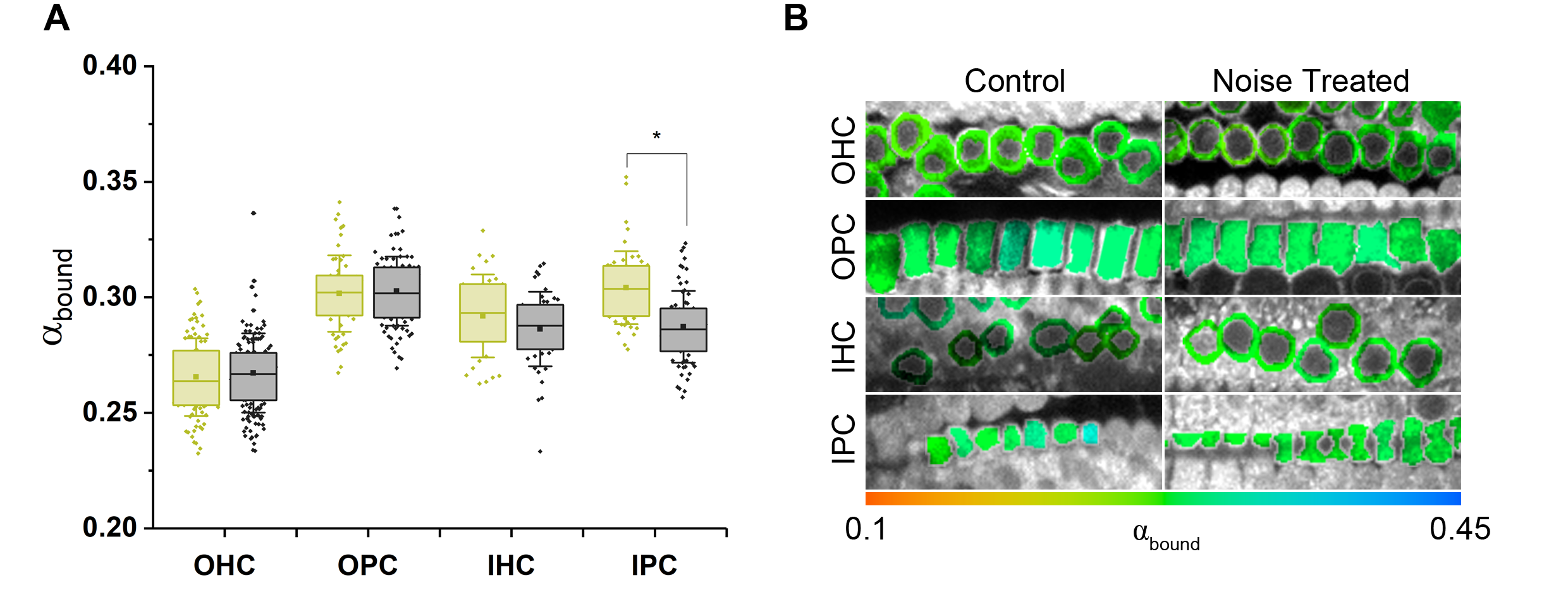


**Figure S4:** values obtained from the experiments summarised in Figure 1. Noise exposure caused to decrease in IPCs only.


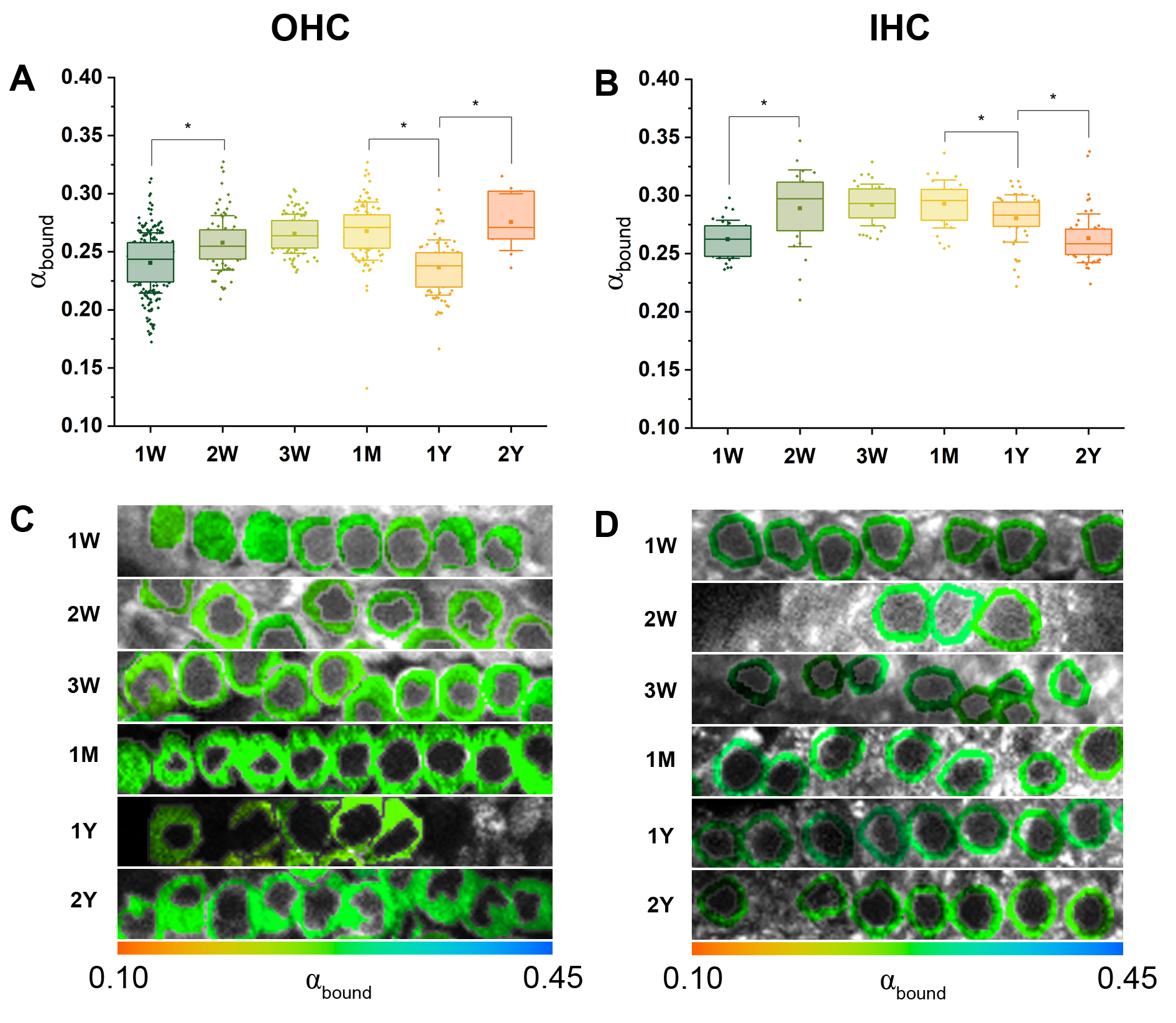


**Figure S5:** Changes in in HCs during development and ageing obtained from the experiments summarised in Figure 3.


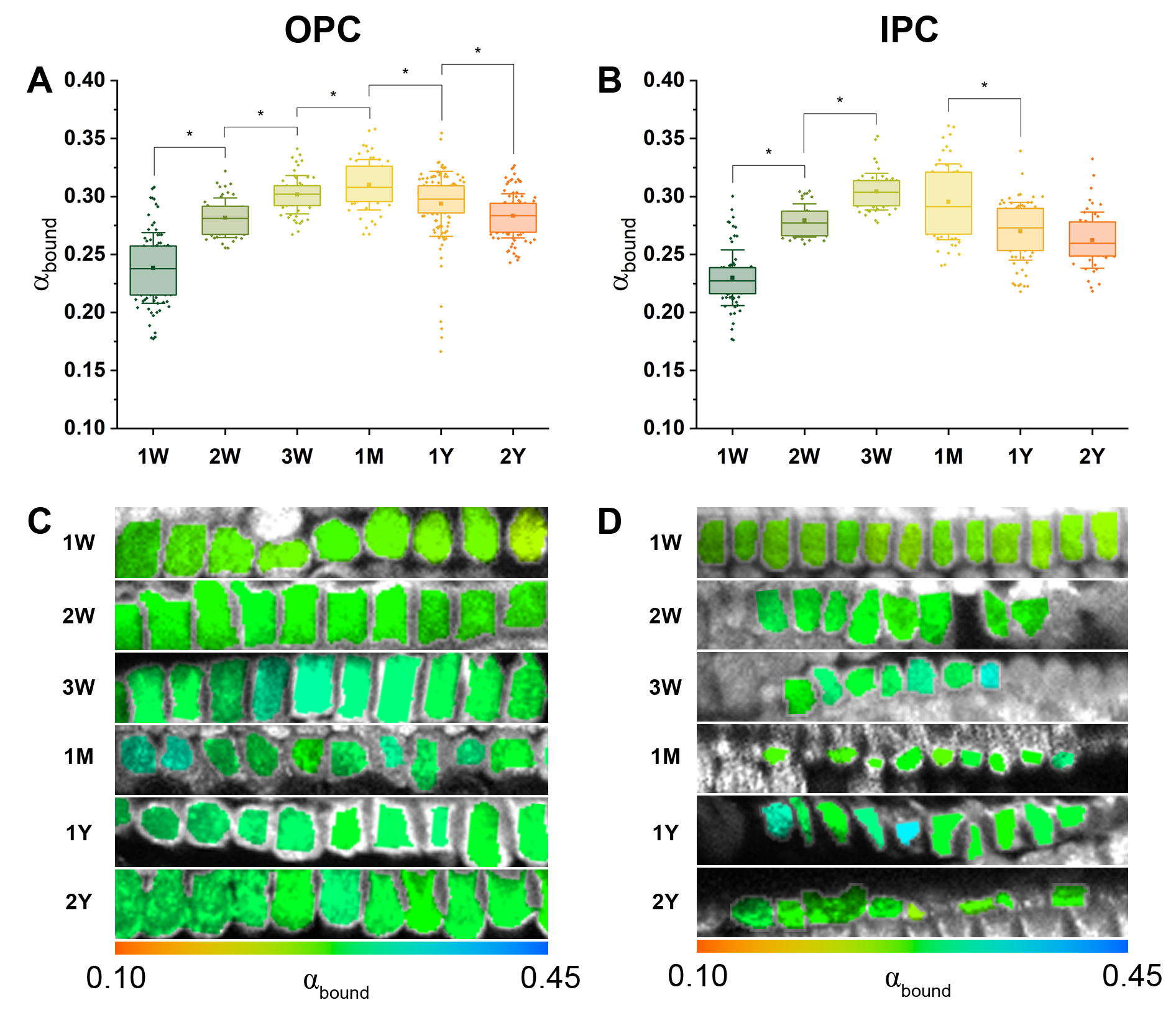


**Figure S6:** Changes in in PCs during development and ageing obtained from the experiments summarised in Figure 3.


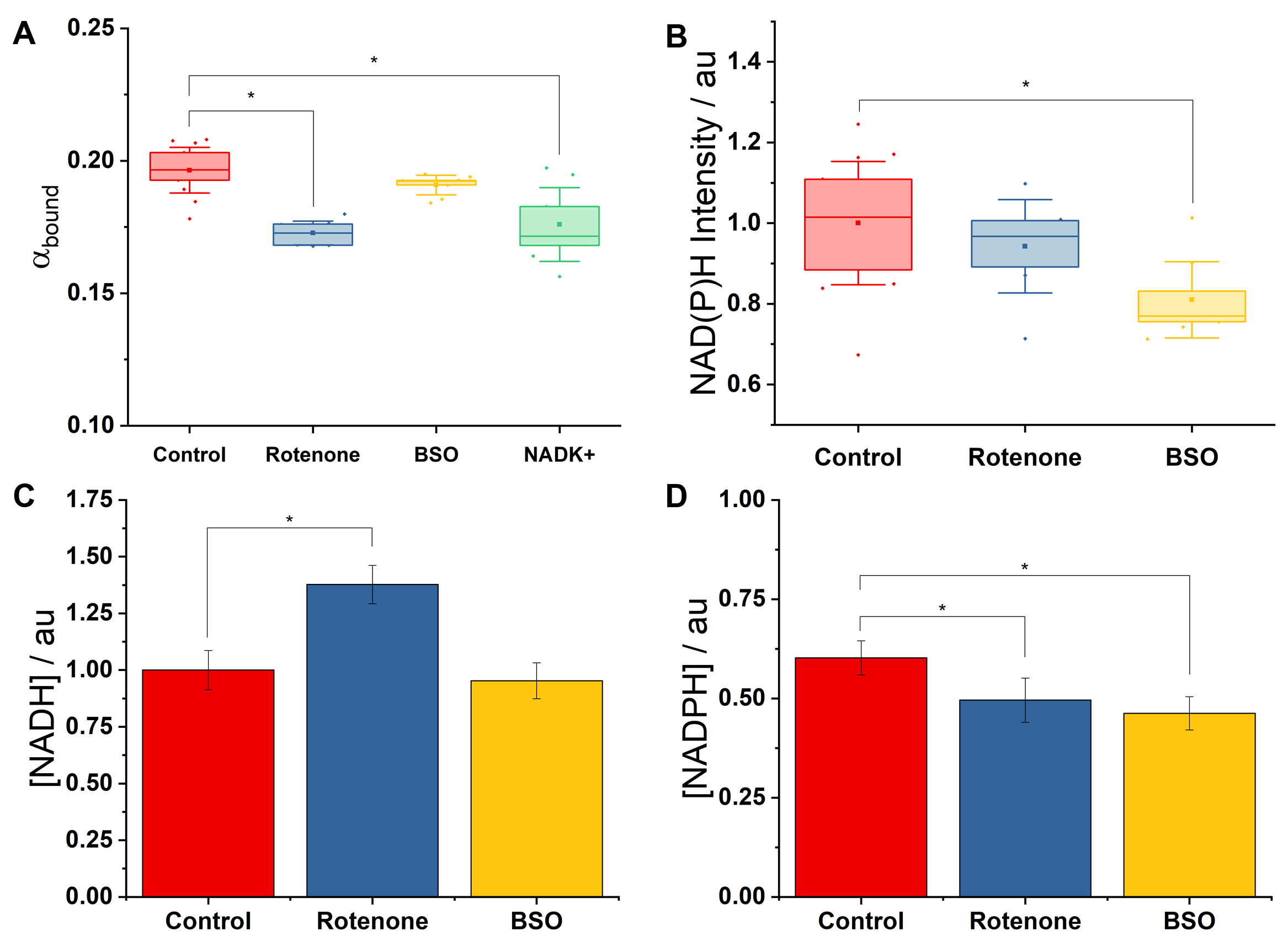


**Figure S7:** Changes in and NAD(P)H fluorescence intensity in HEK293 cells following 24 hour treatments to increase oxidative stress by two separate mechanisms (200 nM rotenone and 100μM BSO), alongside the corresponding calculated changes in NADH and NADPH concentration.


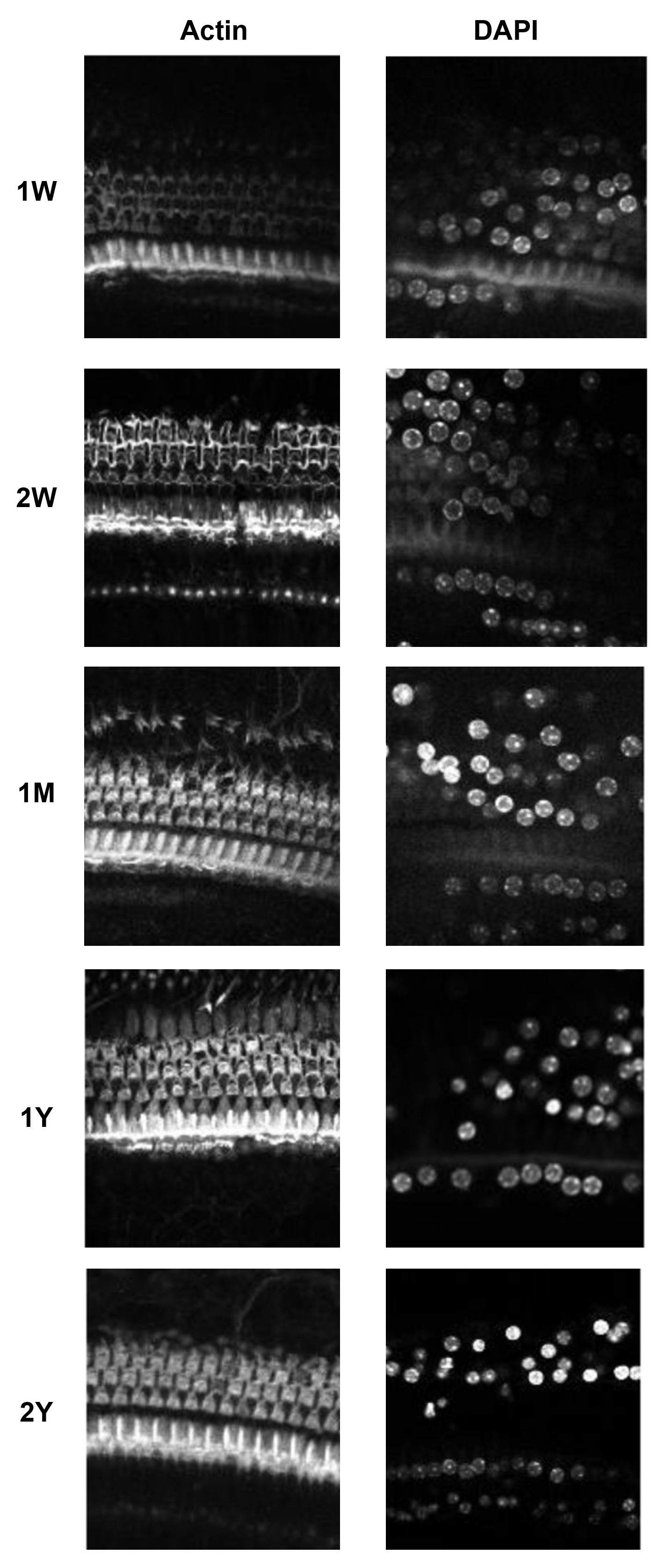


**Figure S8:** Example immunostaining to confirm the viability of the cochlea preparations at each age.

**Supplementary references**

1. Blacker, T. S. *et al.* Separating NADH and NADPH fluorescence in live cells and tissues using FLIM. *Nat. Commun.* **5**, 3936 (2014).

2. Enderlein, J. & Erdmann, R. Fast fitting of multi-exponential decay curves. *Opt. Commun.* **134**, 371–378 (1997).

3. Skala, M. C. *et al.* In vivo multiphoton fluorescence lifetime imaging of protein-bound and free nicotinamide adenine dinucleotide in normal and precancerous epithelia. *J. Biomed. Opt.* **12**, 24014 (2007).

4. Bird, D. K. *et al.* Metabolic Mapping of MCF10A Human Breast Cells via Multiphoton Fluorescence Lifetime Imaging of the Coenzyme NADH. *Cancer Res.* **65**, 8766–8773 (2005).

5. Pate, K. T. *et al.* Wnt signaling directs a metabolic program of glycolysis and angiogenesis in colon cancer. *EMBO J.* **33**, 1454–1473 (2014).

6. Guo, H.-W. *et al.* Correlation of NADH fluorescence lifetime and oxidative phosphorylation metabolism in the osteogenic differentiation of human mesenchymal stem cell. *J. Biomed. Opt.* **20**, 017004 (2015).

7. Vignier, N. *et al.* Rescue of biosynthesis of nicotinamide adenine dinucleotide protects the heart in cardiomyopathy caused by lamin A/C gene mutation. *Hum. Mol. Genet.* **27**, 3870–3880 (2018).

8. Nikiforov, A., Kulikova, V. & Ziegler, M. The human NAD metabolome: Functions, metabolism and compartmentalization. *Crit. Rev. Biochem. Mol. Biol.* **50**, 284–97 (2015).

9. Schaefer, P. M. *et al.* Mitochondrial matrix pH as a decisive factor in neurometabolic imaging. *Neurophotonics* **4**, 45004 (2017).

10. Selivanov, V. A. *et al.* The role of external and matrix pH in mitochondrial reactive oxygen species generation. *J. Biol. Chem.* (2008).
